# USP27X, a new player on EMT and fibroblast activation

**DOI:** 10.18632/oncotarget.26410

**Published:** 2018-12-04

**Authors:** Víctor M. Díaz

**Affiliations:** Víctor M. Díaz: Programa de Recerca en Càncer, Institut Hospital del Mar d’Investigacions Mèdiques (IMIM), Unidad Asociada CSIC, Barcelona, Spain; Departament de Ciències Experimentals i de la Salut, Universitat Pompeu Fabra (UPF), Barcelona, Spain

**Keywords:** USP27X, Snail1, EMT, cancer, metastasis

**Figure 1:**
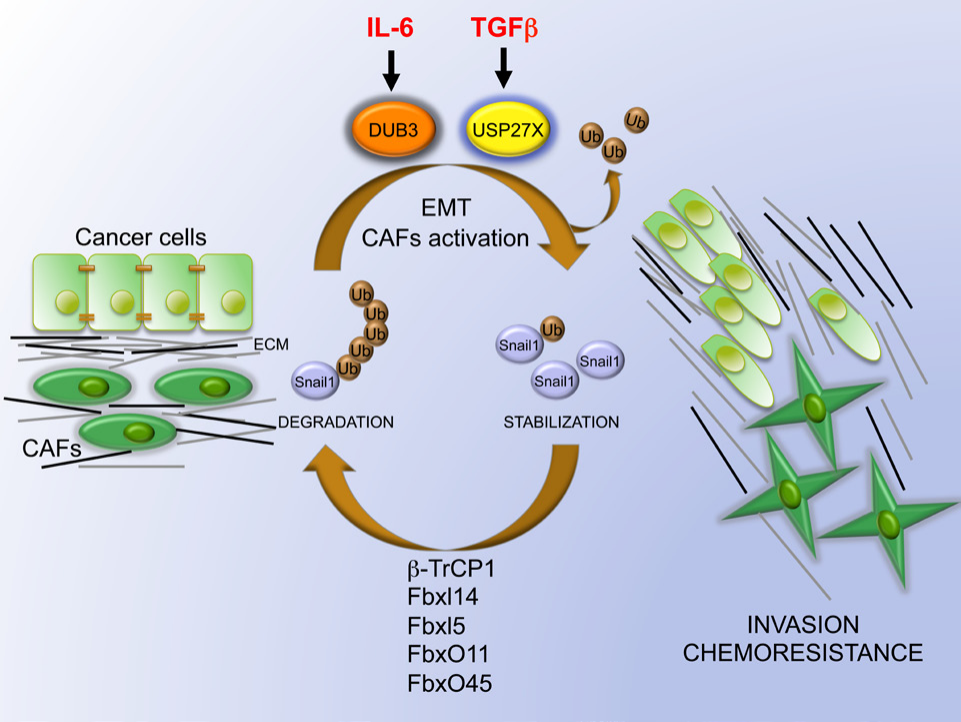
Role of USP27X in mediating EMT and CAF activation Snail1 is highly unstable in cancer epithelial cells and non-activated fibroblasts because it is continuously ubiquitinated by several E3-ubiquitin ligases (β-TrCP1, Fbxl14, Fbxl5, FbxO11 and FbxO45). TGFβ enhances USP27X expression that deubiquitinates and stabilizes Snail1, which in turn induces EMT in cancer cells and activation of CAFs. Similarly, IL-6 activates Dub3 contributing to Snail1 stabilization. Once activated, CAFs increase extracellular matrix (ECM) deposition and alignment, acting as a guide for cell invasion, metastasis formation and increased chemoresistance. Ub, ubiquitin.

The epithelial-mesenchymal transition (EMT) shifts epithelial cells towards a malignant mesenchymal phenotype, enhancing their invasiveness and chemoresistance. Cancer cells initiate an “EMT program” for their metastatic dissemination and this in turn is governed by several transcription factors including Snail1, Slug (Snail2), Twist and Zeb that repress epithelial and activate mesenchymal transcriptional signatures [1]. Snail1 promotes tumor invasion, resistance to antineoplastic drugs, acquisition of cancer stem cell characteristics and metabolism reprogramming [1]. Besides, Snail1 controls activation of cancer-activated fibroblasts (CAFs) [2] (Figure 1). Accordingly, the expression of Snail1 is observed in the tumor-stroma interface of colorectal tumors [3] and in triple-negative breast cancer (TNBC), the most lethal and aggressive subtype [4]. Unfortunately, since Snail1 role was discovered in cancer cells, it has represented an elusive molecular target for therapeutic intervention. With this goal we have pointed to the Achilles’ heel of Snail1, which is its remarkable protein instability [5].

An extensive work has been done during the last years describing Snail1 lability in epithelial cells pushed by the action of several E3-ubiquitin ligases [6] (Figure 1). Cancer cells manage to counteract Snail1 protein degradation by activating signaling pathways, such us the Wnt/AKT/GSK3β, the Notch/β-catenin/AKT/GSK3β and others initiated by Hypoxia, DNA damage and cell stress [6]. Pathological Snail1 stabilization requires the specific down-regulation of one or several E3 ligases like FBXL14, the inactivation of kinases such us GSK3β required for β-TrCP1 degradation or the action of kinases enhancing Snail1 stability [6]. We have focused in an alternative scenario, on the enzymes that removed the ubiquitination mark, the deubiquitinases (DUBs). These DUBs are currently at the main focus of cancer research because are commonly deregulated in tumors [7]. To tackle the issue of DUBs action on Snail1 stability, we have performed an unbiased luminescent siRNA screening and identified USP27X as a bona fide DUB for Snail1. USP27X was validated by an extensive battery of biochemical assays testing the physical interaction with Snail1 and the capability to stabilize and to deubiquitinate Snail1 [5] (Figure 1).

USP27X is detected in cancer cell lines and tumors with a 72 KDa molecular weight, higher than the expected because its translation occurs upstream of the canonical AUG codon [8]. This long form is enriched in the nucleus but also present in the cytosol. Although the existence of a protein translated from the canonical AUG has not been formally proven, a shorter form is detected in the cytosol of cancer cells, suggesting that different forms of USP27X might have specific roles. Functionally, USP27X is closely related to USP22 and to USP51, DUBs targeting monoubiquitinated H2B [8]. USP22 forms part of the SAGA complex and requires the participation of cofactors like ATXN7L3 and ENY2 to deubiquitinate H2B, while USP27X functions independently of SAGA but also needs ATXN7L3 and ENY2 to act on H2B [8]. The similar participation of cofactors has not been investigated for USP27X action on Snail1 or others substrates. When over-expressed, USP22 interacts with and stabilizes Snail1; however, the fact USP22 cannot compensate the lack of USP27X (in terms of Snail1 stability) suggest USP27X has a predominant role in Snail1 stabilization [5]. Therefore, several questions still need to be responded: 1) Might different forms of USP27X have a differential role in EMT? 2) Which is the relationship between USP27X and USP22? 3) Does USP27X require specific cofactors to deubiquitinate Snail1? The answer to these questions will be essential to have a deeper knowledge of USP27X role on Snail1 function.

USP27X knock-down (KO) cells showed impaired cell invasion and metastasis formation, being both effects partially rescued by Snail1 [5]. However, USP27X KO cells also showed decreased tumor growth, which was not directly rescued by Snail1 expression. The lower potential to extravasate and/or colonize lungs showed by USP27X KO cells suggests a predominant role in USP27X-Snail axis in metastasis formation. On the other hand, effects on cell proliferation may be explained by the action of USP27X on alternative substrates [8]. In any case, USP27X remarkably affects tumor development in multiple steps of cancer progression. EMT also shows a remarkable effect on chemotherapy resistance [6]. Snail1 becomes upregulated upon cisplatin treatment of breast cancer cells in part as a consequence of USP27X action, which results in increased apoptosis resistance. Inhibition of USP27X enhanced cell death promoted by exposition to cisplatin [5], opening the possibility to use small molecules against this enzyme to restore or potentiate chemosensitivy to cisplatin or other drugs.

Recently, Dub3 was also described as a deubiquitinase of Snail1 [9]. Whereas USP27X only targets Snail1 and not Snail2, Dub3 acts on both Snail1 and Snail2 and also Twist, suggesting a broader spectrum activity. In addition, USP27X is under the control of TGFβ, while Dub3 was up-regulated by IL-6-mediated inflammation (Figure 1) [5, 9]. TGFβ has been shown to induce cancer-associated (CAFs) activation [2] and the role of USP27X is particularly relevant in this process [5] (Figure 1). Activated CAFs secrete metalloproteinases enhancing cell invasion and act as a guide for invading epithelial tumoral cells during metastasis formation (Figure 1). Similarly, carcinoma-derived Il-6 also enhances fibroblast activation [10]. So both USP27X and Dub3 may have a predominant role regulating the paracrine interplay between epithelial carcinoma cells and CAFs. In this regard, drugs blocking USP27X and/or Dub3 activities will be a useful way to interfere the connection between CAFs and cancer cells.

In summary, DUBs targeting Snail1 are an attractive target for chemotherapy. The description of USP27X as a TGFβ-activated DUB and its role on EMT provides new clues to specifically counteract the pathological stabilization of Snail1. The development of selective USP27X inhibitors will offer new possibilities to fight against metastasis formation.
